# Effects of SARS-CoV-2 Alpha, Beta, and Delta variants, age, vaccination, and prior infection on infectiousness of SARS-CoV-2 infections

**DOI:** 10.3389/fimmu.2022.984784

**Published:** 2022-09-13

**Authors:** Suelen H. Qassim, Mohammad R. Hasan, Patrick Tang, Hiam Chemaitelly, Houssein H. Ayoub, Hadi M. Yassine, Hebah A. Al-Khatib, Maria K. Smatti, Hanan F. Abdul-Rahim, Gheyath K. Nasrallah, Mohamed Ghaith Al-Kuwari, Abdullatif Al-Khal, Peter Coyle, Imtiaz Gillani, Anvar Hassan Kaleeckal, Riyazuddin Mohammad Shaik, Ali Nizar Latif, Einas Al-Kuwari, Andrew Jeremijenko, Adeel A. Butt, Roberto Bertollini, Hamad Eid Al-Romaihi, Mohamed H. Al-Thani, Laith J. Abu-Raddad

**Affiliations:** ^1^ Infectious Disease Epidemiology Group, Weill Cornell Medicine-Qatar, Cornell University, Doha, Qatar; ^2^ World Health Organization Collaborating Centre for Disease Epidemiology Analytics on HIV/AIDS, Sexually Transmitted Infections, and Viral Hepatitis, Weill Cornell Medicine–Qatar, Cornell University, Qatar Foundation – Education City, Doha, Qatar; ^3^ Department of Population Health Sciences, Weill Cornell Medicine, Cornell University, New York, NY, United States; ^4^ Department of Pathology, Sidra Medicine, Doha, Qatar; ^5^ Mathematics Program, Department of Mathematics, Statistics, and Physics, College of Arts and Sciences, Qatar University, Doha, Qatar; ^6^ Biomedical Research Center, QU Health, Qatar University, Doha, Qatar; ^7^ Department of Biomedical Science, College of Health Sciences, QU Health, Qatar University, Doha, Qatar; ^8^ Department of Public Health, College of Health Sciences, QU Health, Qatar University, Doha, Qatar; ^9^ Strategy Planning and Health Intelligence Primary Health Care Corporation, Doha, Qatar; ^10^ Hamad Medical Corporation, Doha, Qatar; ^11^ Wellcome-Wolfson Institute for Experimental Medicine, Queens University, Belfast, United Kingdom; ^12^ Department of Medicine, Weill Cornell Medicine, Cornell University, New York, NY, United States; ^13^ Ministry of Public Health, Doha, Qatar

**Keywords:** COVID-19, SARS-CoV-2 variant, vaccine, reinfection, breakthrough infection, immunity, epidemiology, PCR

## Abstract

In 2021, Qatar experienced considerable incidence of SARS-CoV-2 infection that was dominated sequentially by the Alpha, Beta, and Delta variants. Using the cycle threshold (Ct) value of an RT-qPCR-positive test to proxy the inverse of infectiousness, we investigated infectiousness of SARS-CoV-2 infections by variant, age, sex, vaccination status, prior infection status, and reason for testing in a random sample of 18,355 RT-qPCR-genotyped infections. Regression analyses were conducted to estimate associations with the Ct value of RT-qPCR-positive tests. Compared to Beta infections, Alpha and Delta infections demonstrated 2.56 higher Ct cycles (95% CI: 2.35-2.78), and 4.92 fewer cycles (95% CI: 4.67- 5.16), respectively. The Ct value declined gradually with age and was especially high for children <10 years of age, signifying lower infectiousness in small children. Children <10 years of age had 2.18 higher Ct cycles (95% CI: 1.88-2.48) than those 10-19 years of age. Compared to unvaccinated individuals, the Ct value was higher among individuals who had received one or two vaccine doses, but the Ct value decreased gradually with time since the second-dose vaccination. Ct value was 2.07 cycles higher (95% CI: 1.42-2.72) for those with a prior infection than those without prior infection. The Ct value was lowest among individuals tested because of symptoms and was highest among individuals tested as a travel requirement. Delta was substantially more infectious than Beta. Prior immunity, whether due to vaccination or prior infection, is associated with lower infectiousness of breakthrough infections, but infectiousness increases gradually with time since the second-dose vaccination.

## Introduction

The severe acute respiratory syndrome coronavirus 2 (SARS-CoV-2) pandemic continues with progressive viral evolution more than two years after it first emerged (1). Between January 18, 2021 and May 31, 2021, Qatar experienced a SARS-CoV-2 Alpha (2) (B.1.1.7) variant wave (3) that was immediately followed by a Beta (2) (B.1.351) variant wave (4). Starting in June 2021, the Delta (2) (B.1.617.2) variant dominated a prolonged low-incidence phase that persisted until November of 2021 (5–7). We investigated the effects of SARS-CoV-2 variant, age, vaccination, and prior infection on infectiousness of SARS-CoV-2 infections utilizing the threshold cycle (Ct) values from SARS-CoV-2 reverse transcription quantitative polymerase chain reaction (RT-qPCR) as the proxy for infectiousness.

In real-time PCR, the RT-qPCR Ct value refers to the number of amplification cycles at which fluorescent signals generated in a reaction crosses a pre-set, detection threshold. The higher the concentration of PCR targets in the reaction, the earlier the cycle it is detected and the lower the Ct value it generates (8). In RT-qPCR assays for detection of viruses such as SARS-CoV-2, Ct values are inversely proportional to the viral load, and are strongly correlated to the cultivability of the virus (9, 10). Therefore, RT-qPCR Ct values have been widely used to proxy SARS-CoV-2 infectiousness with lower Ct values indicating higher infectiousness and higher Ct values indicating lower infectiousness (10–15). Earlier studies found that lower Ct values (or higher viral loads) in samples of SARS-CoV-2 cases were associated with higher transmission rate and transmission risk, such as in studies of household transmission and among contacts of cases (11, 13). We assessed differences in Ct values in a random sample of 18,355 RT-qPCR-genotyped SARS-CoV-2 infections in relation to variant status, age, sex, vaccination status, prior infection status, and reason for testing.

## Methods

### Study population, data sources, and study design

This cross sectional study was conducted in the resident population of Qatar, applying a methodology used recently to investigate effects of the BA.1/BA.2 subvariant, vaccination, and prior infection on infectiousness of SARS-CoV-2 Omicron (2) (B.1.1.529) infections (16). Similarly, several effects on the infectiousness of SARS-CoV-2 infections were investigated including pre-Omicron variants (Alpha, Beta, and Delta), mRNA COVID-19 vaccination status [BNT162b2 (Pfizer-BioNTech) (17) and mRNA-1273 (Moderna) (18)], prior infection status, reason for RT-qPCR testing, study-period month of the RT-qPCR test (to account for the evolving phase of SARS-CoV-2 incidence), and demographic factors, including sex, age, and nationality.

The present study was conducted on a sample of 18,355 SARS-CoV-2 RT-qPCR-positive swabs that were collected randomly on a weekly basis from among all RT-qPCR-confirmed infections in Qatar between March 23, 2021 and November 6, 2021. These documented infections were RT-qPCR genotyped as part of a national project for surveillance of SARS-CoV-2 variants in Qatar (7, 19–21). Details of laboratory methods for RT-qPCR testing and variant ascertainment are provided in Section 1 of the **Supplementary Material**. Coronavirus disease 2019 (COVID-19) laboratory testing, vaccination, clinical infection, and demographic data for this population were extracted from the national, federated SARS-CoV-2 databases, which include all RT-qPCR testing, reason for RT-qPCR testing, COVID-19 vaccinations, and related demographic details since the start of the pandemic. Further description of Qatar’s national COVID-19 databases has been reported previously (4, 6, 7, 15, 22).

Every SARS-CoV-2 RT-qPCR test conducted in Qatar is classified based on the reason for testing (clinical symptoms, contact tracing, surveys or random testing campaigns, individual requests, routine healthcare testing, pre-travel, at port of entry, or other). RT-qPCR testing is performed at a mass scale and most infections are diagnosed not for appearance of symptoms, but because of routine testing (6). Qatar has unusually young, diverse demographics, in that only 9% of its residents are ≥50 years of age, and 89% are expatriates from over 150 countries (22, 23). Nearly all individuals were vaccinated in Qatar; however, vaccinations performed elsewhere were recorded in the health system at the port of entry upon arrival to Qatar, per national requirements.

For standardization of RT-qPCR Ct values, we analyzed only RT-qPCR-confirmed infections diagnosed with the TaqPath COVID-19 Combo Kit [Thermo Fisher Scientific, USA (24)]. For each individual, all RT-qPCR-positive swabs during the study period were included, provided that at least 90 days had elapsed between two consecutive positive swabs to avoid inclusion of positive results from the same infectious episode (25, 26). A summary measure was derived for the primary outcome, the RT-qPCR Ct value (9), by averaging Ct values of the N, ORF1ab, and S gene targets. This average Ct value was used as the dependent variable in all analyses.

Both vaccination status and prior infection status were ascertained at the time of the RT-qPCR test. Vaccination status was defined by the number of administered vaccine doses and months elapsed since the last vaccine dose, with one month defined as 30 days. Only individuals vaccinated with BNT162b2 (17) or mRNA-1273 (18) vaccines were included in the analyses, as these have been the vaccines of choice in the COVID-19 immunization program in Qatar (27–29). Rare occurrences of mixed vaccination regimens were excluded. Nearly all vaccinated persons received their second vaccine dose per protocol (17, 18) within 30 days of first dose. History of prior infection was defined as an RT-qPCR-positive test that occurred ≥90 days before the study RT-qPCR-positive test (4, 5, 30, 31). An RT-qPCR-positive test that occurred <90 days prior to the study RT-qPCR-positive test was not considered a prior infection, but was considered a category of its own. This is because the prior RT-qPCR-positive test and the study RT-qPCR-positive test may both reflect the same infection (25, 26). A small number of RT-qPCR tests that had no recorded Ct value were excluded from the analysis, but these constituted only 0.5% of all RT-qPCR-genotyped infections. Otherwise, data for the remaining study variables were complete.

### Oversight

Hamad Medical Corporation and Weill Cornell Medicine-Qatar Institutional Review Boards approved this retrospective study with a waiver of informed consent. The study was reported following the Strengthening the Reporting of Observational Studies in Epidemiology (STROBE) guidelines. The STROBE checklist is found in **Supplementary Table 1**.

### Statistical analysis

Frequency distributions and measures of central tendency were used to describe the study population with respect to *a priori* determined factors. These included SARS-CoV-2 variant, vaccination status (factoring dose number and months since vaccination), prior infection status, reason for RT-qPCR testing, study-period month of the RT-qPCR test, and demographic factors, age, sex, and nationality.

Association of each of these factors with Ct value was assessed using univariable linear regression analyses. Unadjusted β coefficients, 95% confidence intervals (CIs), and the F-test of overall covariate significance were reported. Adjusted β coefficients and associated 95% CIs and p-values were estimated using multivariable linear regression analyses that included all covariates in the model.

Two-sided p-value <0.05 indicated statistical significance. Interactions were not considered. Statistical analyses were conducted in STATA/SE version 16 (32).

## Results

**Figure 1:**
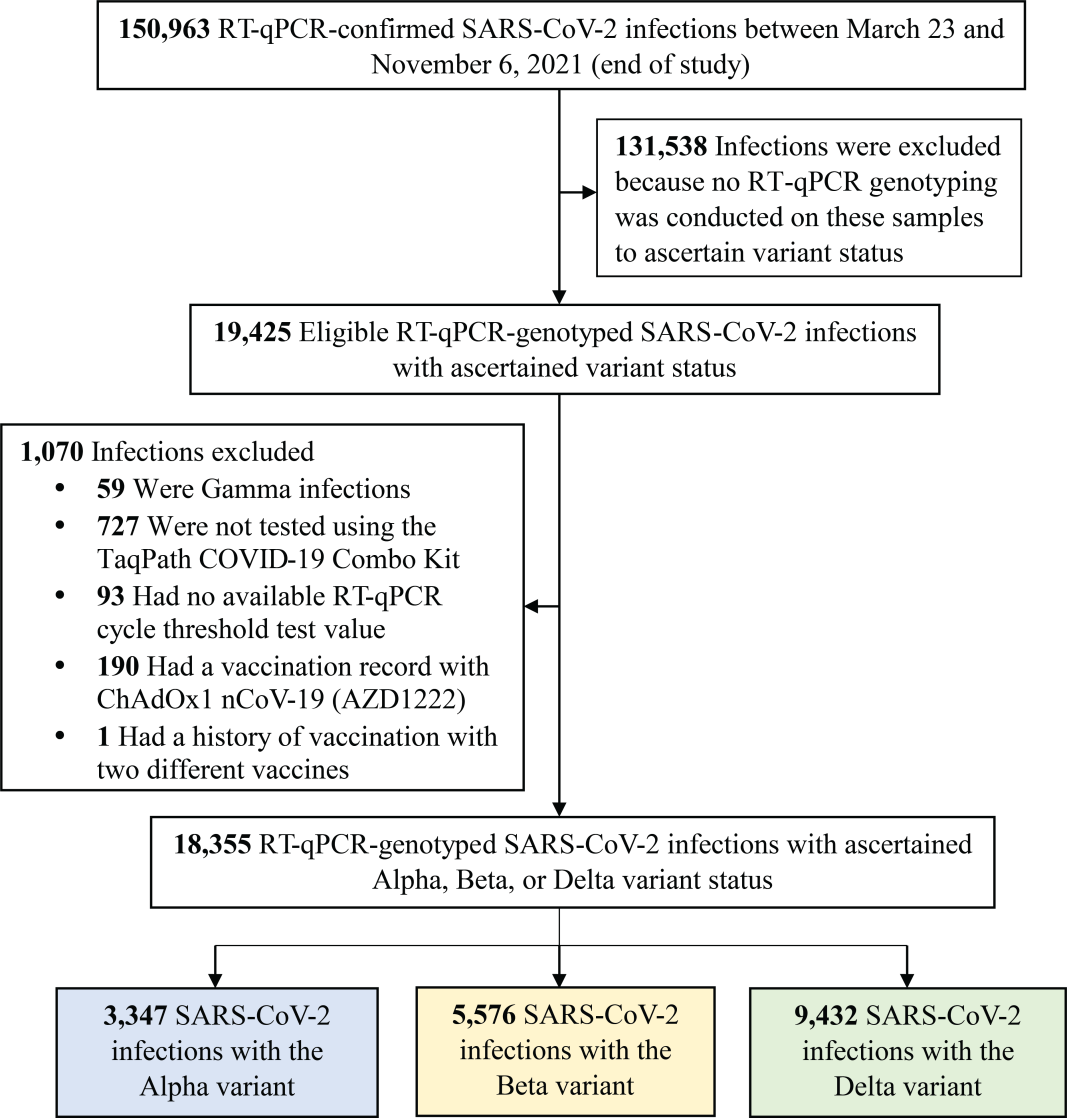
Flowchart describing the population selection process for investigating infectiousness of SARS-CoV-2 Alpha, Beta and Delta infections. Abbreviations: COVID-19, coronavirus disease 2019; RT-qPCR, real-time reverse-transcription polymerase chain reaction; SARS-CoV-2, severe acute respiratory syndrome coronavirus 2.

**Figure 1** shows the process of selecting the study population and **Table 1** describes study population characteristics. This was a national study involving a random sample of 18,355 RT-qPCR-confirmed SARS-CoV-2 infections. Therefore, the study population is broadly representative of the population of Qatar. The sample included 3,347 (18.2%) Alpha infections, 5,576 (30.4%) Beta infections, and 9,432 (51.4%) Delta infections (**Table 1**).

**Table 1:** Characteristics of the 18,355 RT-qPCR-genotyped SARS-CoV-2 infections between March 23 and November 6, 2021.

Characteristics	Total sample	Alpha infections	Beta infections	Delta infections
	N (%)	N (%)	N (%)	N (%)
Total N	18,355	3,347 (18.2)	5,576 (30.4)	9,432 (51.4)
Demographic characteristics				
Median age (IQR) — years	32 (22-41)	33 (25-41)	33 (25-42)	31 (18-40)
Age group in years — no. (%)				
<10	1,956 (10.7)	286 (8.5)	424 (7.6)	1,246 (13.2)
10-19	2,042 (11.1)	277 (8.3)	499 (8.9)	1,266 (13.4)
20-29	3,670 (20.0)	749 (22.4)	1,104 (19.8)	1,817 (19.3)
30-39	5,624 (30.6)	1,104 (33.0)	1,866 (33.5)	2,654 (28.1)
40-49	3,263 (17.8)	627 (18.7)	1,074 (19.3)	1,562 (16.6)
50-59	1,240 (6.8)	210 (6.3)	431 (7.7)	599 (6.4)
60-69	426 (2.3)	73 (2.2)	134 (2.4)	219 (2.3)
70-79	95 (0.5)	14 (0.4)	31 (0.6)	50 (0.5)
80+	39 (0.2)	7 (0.2)	13 (0.2)	19 (0.2)
Sex				
Female	6,805 (37.1)	1,114 (33.3)	1,813 (32.5)	3,878 (41.1)
Male	11,550 (62.9)	2,233 (66.7)	3,763 (67.5)	5,554 (58.9)
Nationality^†^				
Bangladeshi	1,077 (5.9)	240 (7.2)	470 (8.4)	367 (3.9)
Egyptian	1,098 (6.0)	115 (3.4)	311 (5.6)	672 (7.1)
Filipino	1,555 (8.5)	340 (10.2)	582 (10.4)	633 (6.7)
Indian	3,873 (21.1)	855 (25.5)	1,355 (24.3)	1,663 (17.6)
Nepalese	1,084 (5.9)	225 (6.7)	459 (8.2)	400 (4.2)
Pakistani	1,017 (5.5)	223 (6.7)	279 (5.0)	515 (5.5)
Qatari	3,903 (21.3)	555 (16.6)	850 (15.2)	2,498 (26.5)
Sri Lankan	488 (2.7)	106 (3.2)	216 (3.9)	166 (1.8)
Sudanese	394 (2.1)	92 (2.7)	108 (1.9)	194 (2.1)
Other nationalities^‡^	3,866 (21.1)	596 (17.8)	946 (17.0)	2,324 (24.6)
RT-qPCR test characteristics				
Reason for RT-qPCR testing				
Clinical suspicion	6,499 (35.4)	1,067 (31.9)	2,477 (44.4)	2,955 (31.3)
Contact tracing	2,261 (12.3)	404 (12.1)	775 (13.9)	1,082 (11.5)
Healthcare routine testing	532 (2.9)	115 (3.4)	218 (3.9)	199 (2.1)
Survey	2,770 (15.1)	552 (16.5)	938 (16.8)	1,280 (13.6)
Port of entry	3,245 (17.7)	522 (15.6)	291 (5.2)	2,432 (25.8)
Pre-travel	1,243 (6.8)	201 (6.0)	225 (4.0)	817 (8.7)
Individual request	1,620 (8.8)	435 (13.0)	603 (10.8)	582 (6.2)
Other	185 (1.0)	51 (1.5)	49 (0.9)	85 (0.9)
RT-qPCR test study-period month				
23 March-21 April, 2021	5,185 (28.2)	1,415 (42.3)	3,564 (63.9)	206 (2.2)
22 April-21 May, 2021	2,502 (13.6)	736 (22.0)	1,262 (22.6)	504 (5.3)
22 May-20 June, 2021	1,287 (7.0)	597 (17.8)	226 (4.1)	464 (4.9)
21 June-20 July, 2021	1,263 (6.9)	258 (7.7)	111 (2.0)	894 (9.5)
21 July-19 August, 2021	3,647 (19.9)	253 (7.6)	326 (5.8)	3,068 (32.5)
20 August-18 September, 2021	2,807 (15.3)	88 (2.6)	87 (1.6)	2,632 (27.9)
19 September-18 October, 2021	976 (5.3)	0 (0.0)	0 (0.0)	976 (10.3)
19 October-06 November, 2021	688 (3.7)	0 (0.0)	0 (0.0)	688 (7.3)
Vaccine and natural immunity				
Vaccination status				
Unvaccinated	12,865 (70.1)	2,760 (82.5)	4,429 (79.4)	5,676 (60.2)
One dose	1,053 (5.7)	239 (7.1)	582 (10.4)	232 (2.5)
Two doses				
<3 months before the RT-qPCR test	1,388 (7.6)	258 (7.7)	449 (8.1)	681 (7.2)
3-<6 months before the RT-qPCR test	2,469 (13.5)	86 (2.6)	107 (1.9)	2,276 (24.1)
6-<9 months before the RT-qPCR test	567 (3.1)	3 (0.1)	7 (0.1)	557 (5.9)
≥9 months before the RT-qPCR test	4 (0.0)	0 (0.0)	0 (0.0)	4 (0.0)
Three doses				
≤1 month before the RT-qPCR test	5 (0.0)	1 (0.0)	2 (0.0)	2 (0.0)
>1 month before the RT-qPCR test	4 (0.0)	0 (0.0)	0 (0.0)	4 (0.0)
Prior SARS-CoV-2 infection				
Never	15,785 (86.0)	2,540 (75.9)	4,911 (88.1)	8,334 (88.4)
<90 days before the study RT-qPCR test^§^	2,362 (12.9)	772 (23.1)	605 (10.9)	985 (10.4)
Prior infection^¶^	208 (1.1)	35 (1.0)	60 (1.1)	113 (1.2)

IQR, interquartile range; RT-qPCR, real-time reverse-transcription polymerase chain reaction; SARS-CoV-2, severe acute respiratory syndrome coronavirus 2.

^†^Nationalities were chosen to represent the most populous groups in Qatar.

^‡^These comprise 99 other nationalities in Qatar.

^§^An RT-qPCR-positive test that occurred <90 days before the study RT-qPCR-positive test was included as a separate category in the analysis and was not considered a prior infection. This RT-qPCR-positive test and the study RT-qPCR-positive test may both reflect the same prolonged infection.

^¶^Prior infection was defined as an RT-qPCR-positive test that occurred ≥90 days before the RT-qPCR-positive test that is included in the study.

Compared to Beta infections, Alpha and Delta infections were associated with 2.56 higher Ct cycles (95% CI: 2.35-2.78), and 4.92 fewer cycles (95% CI: 4.67- 5.16) (**Table 2**), respectively, indicating the highest infectiousness for the Delta variant.

**Table 2:** Associations with RT-qPCR Ct value among the 18,355 RT-qPCR-genotyped SARS-CoV-2 infections between March 23 and November 6, 2021.

Characteristics	RT-qPCR Ct value	Univariable analysis		F-test^*^	Multivariable analysis^†^	
	Mean (SD)	β coefficient [95% CI]	p-value	p-value	β coefficient [95% CI]	p-value
Age group in years				<0.001		
10-19^‡^	23.23 (5.39)	Ref.			Ref.	
<10	25.51 (5.06)	2.28 [1.93, 2.63]	<0.001		2.18 [1.88, 2.48]	<0.001
20-29	23.74 (5.80)	0.52 [0.21, 0.82]	0.001		-0.24 [-0.51, 0.04]	0.091
30-39	23.63 (5.73)	0.41 [0.12, 0.69]	0.005		-0.39 [-0.65, -0.12]	0.004
40-49	23.43 (5.78)	0.20 [-0.11, 0.51]	0.204		-0.52 [-0.81, -0.23]	<0.001
50-59	23.55 (5.50)	0.33 [-0.07, 0.72]	0.106		-0.44 [-0.80, -0.09]	0.014
60-69	23.35 (5.31)	0.12 [-0.46, 0.71]	0.677		-0.55 [-1.06, -0.04]	0.036
70-79	22.70 (5.23)	-0.52 [-1.68, 0.63]	0.375		-1.01 [-1.99, -0.03]	0.044
80+	22.86 (5.27)	-0.37 [-2.15, 1.42]	0.688		-0.83 [-2.33, 0.68]	0.280
Sex				<0.001		
Female	23.05 (5.48)	Ref.			Ref.	
Male	24.17 (5.72)	1.13 [0.96, 1.29]	<0.001		0.60 [0.45, 0.76]	<0.001
Nationality^§^				<0.001		
Qatari	23.13 (5.31)	Ref.			Ref.	
Bangladeshi	24.34 (5.94)	1.20 [0.82, 1.58]	<0.001		0.55 [0.20, 0.90]	0.002
Egyptian	23.53 (5.38)	0.40 [0.02, 0.77]	0.039		0.23 [-0.09, 0.56]	0.161
Filipino	23.25 (5.72)	0.11 [-0.22, 0.45]	0.496		0.19 [-0.12, 0.49]	0.227
Indian	24.40 (5.96)	1.27 [1.02, 1.52]	<0.001		0.65 [0.41, 0.90]	<0.001
Nepalese	24.31 (5.80)	1.18 [0.80, 1.56]	<0.001		0.85 [0.49, 1.20]	<0.001
Pakistani	24.23 (5.69)	1.09 [0.71, 1.48]	<0.001		0.24 [-0.10, 0.59]	0.170
Sri Lankan	24.18 (5.70)	1.05 [0.52, 1.58]	<0.001		0.29 [-0.17, 0.75]	0.217
Sudanese	23.79 (5.06)	0.66 [0.07, 1.24]	0.027		0.64 [0.14, 1.14]	0.012
Other nationalities^¶^	23.51 (5.55)	0.38 [0.13, 0.63]	0.003		0.29 [0.07, 0.50]	0.011
SARS-CoV-2 variant				<0.001		
Beta^**^	23.72 (5.14)	Ref.			Ref.	
Alpha	27.89 (5.65)	4.17 [3.94, 4.39]	<0.001		2.56 [2.35, 2.78]	<0.001
Delta	22.31 (5.21)	-1.41 [-1.59, -1.24]	<0.001		-4.92 [-5.16, -4.67]	<0.001
Reason for RT-qPCR testing				<0.001		
Survey	24.23 (5.52)	Ref.			Ref.	
Clinical suspicion	22.64 (5.42)	-1.59 [-1.84, -1.34]	<0.001		-1.48 [-1.69, -1.26]	<0.001
Contact tracing	23.52 (5.52)	-0.71 [-1.02, -0.40]	<0.001		-0.53 [-0.80, -0.26]	<0.001
Healthcare routine testing	24.20 (5.65)	-0.03 [-0.55, 0.49]	0.913		-0.72 [-1.17, -0.28]	0.001
Port of entry	24.24 (5.66)	0.01 [-0.27, 0.29]	0.934		0.84 [0.59, 1.09]	<0.001
Pre-travel	25.66 (5.66)	1.43 [1.06, 1.81]	<0.001		1.29 [0.97, 1.62]	<0.001
Individual request	24.89 (6.03)	0.66 [0.32, 1.00]	<0.001		0.24 [-0.07, 0.54]	0.125
Other	26.11 (5.62)	1.88 [1.05, 2.71]	<0.001		-1.01 [-1.73, -0.29]	0.006
RT-qPCR test study-period month				<0.001		
23 March-21 April, 2021	23.39 (5.15)	Ref.			Ref.	
22 April-21 May, 2021	24.20 (5.45)	0.82 [0.55, 1.08]	<0.001		1.00 [0.77, 1.23]	<0.001
22 May-20 June, 2021	27.50 (6.46)	4.12 [3.78, 4.46]	<0.001		4.08 [3.76, 4.39]	<0.001
21 June-20 July, 2021	23.07 (5.97)	-0.32 [-0.66, 0.02]	0.068		2.57 [2.23, 2.91]	<0.001
21 July-19 August, 2021	23.29 (5.77)	-0.09 [-0.33, 0.14]	0.442		3.80 [3.52, 4.09]	<0.001
20 August-18 September, 2021	23.96 (5.62)	0.58 [0.32, 0.83]	<0.001		4.97 [4.66, 5.29]	<0.001
19 September-18 October, 2021	23.04 (5.20)	-0.35 [-0.72, 0.03]	0.074		4.46 [4.05, 4.87]	<0.001
19 October-06 November, 2021	21.76 (4.85)	-1.62 [-2.06, -1.18]	<0.001		3.66 [3.20, 4.12]	<0.001
Vaccination status				<0.001		
Unvaccinated	23.98 (5.65)	Ref.			Ref.	
One dose	23.93 (5.71)	-0.05 [-0.40, 0.31]	0.790		0.57 [0.26, 0.87]	<0.001
Two doses						
<3 months before the RT-qPCR test	24.43 (5.86)	0.45 [0.14, 0.77]	0.004		0.86 [0.59, 1.13]	<0.001
3-<6 months before the RT-qPCR test	22.59 (5.40)	-1.39 [-1.63, -1.15]	<0.001		0.08 [-0.17, 0.32]	0.547
6-<9 months before the RT-qPCR test	21.79 (5.12)	-2.19 [-2.66, -1.71]	<0.001		-0.26 [-0.72, 0.19]	0.262
≥9 months before the RT-qPCR test	18.81 (2.22)	-5.17 [-10.68, 0.34]	0.066		-3.23 [-7.89, 1.42]	0.173
Three doses						
≤1 month before the RT-qPCR test	22.99 (5.40)	-0.99 [-5.92, 3.94]	0.693		-1.36 [-5.52, 2.79]	0.520
>1 month before the RT-qPCR test	19.80 (2.97)	-4.18 [-9.69, 1.33]	0.137		-1.83 [-6.47, 2.81]	0.439
Prior SARS-CoV-2 infection				<0.001		
Never	23.14 (5.51)	Ref.			Ref.	
<90 days before the study RT-qPCR test^††^	27.65 (4.93)	4.51 [4.27, 4.74]	<0.001		3.95 [3.73, 4.17]	<0.001
Prior infection^‡‡^	25.88 (5.93)	2.74 [1.99, 3.48]	<0.001		2.07 [1.42, 2.72]	<0.001

CI, confidence interval; Ct, cycle threshold; RT-qPCR, real-time reverse-transcription polymerase chain reaction; Ref., reference; SARS-CoV-2, severe acute respiratory syndrome coronavirus 2; SD, standard deviation.

^*^The two-tailed F-test of the univariable analysis.

^†^RT-qPCR Ct value was adjusted for age-group, sex, nationality, SARS-CoV-2 variant, reason for RT-qPCR test, RT-qPCR test study-period month, vaccination status, and prior SARS-CoV-2 infection.

**^‡^** The 10-19 age group was chosen as a reference, and not the <10 age group, because of the different manifestations of this infection in small children.

^§^Nationalities were chosen to represent the most populous groups on Qatar.

^¶^These comprise 99 other nationalities in Qatar.

^**^Beta was chosen as a reference because of interest in directly comparing Delta to Beta infections.

^††^An RT-qPCR-positive test that occurred <90 days before the study RT-qPCR-positive test was included as a separate category in the analysis and was not considered a prior infection. This RT-qPCR-positive test and the study RT-qPCR-positive test may both reflect the same prolonged infection.

**^‡‡^** Prior infection was defined as an RT-qPCR-positive test that occurred ≥90 days before the RT-qPCR-positive test that is included in the study.

Ct value declined gradually with age and was especially high for children <10 years of age, signifying lower infectiousness of small children. Children <10 years of age had 2.18 higher Ct cycles (95% CI: 1.88-2.48) than those 10-19 years of age (**Table 2**). The 10-19 age group was chosen as a reference, and not the <10 age group, because of the different manifestations of this infection in small children. Males had higher Ct values than females and there were some differences in Ct value by nationality.

Compared to unvaccinated individuals, Ct value was higher among individuals who received one or two vaccine doses (**Table 2**). However, Ct value decreased gradually with time since second-dose vaccination. Very few individuals received a booster dose during the study period (**Table 1**) to allow for estimation of effect of booster vaccination on Ct value. Ct value was 2.07 cycles higher (95% CI: 1.42-2.72) for those with a prior infection compared to those without prior infection.

The Ct value was lowest when testing was performed due to suspicion of infection exposure (**Table 2**), such as appearance of symptoms or recent exposure to an infected person (contact tracing). The Ct value was highest for infections diagnosed because of routine testing for reasons unrelated to infection exposure, such as in a random survey or because of travel requirements. Stratified analyses for Alpha (**Table 3**), Beta (**Table 4**), and Delta (**Table 5**) infections suggested similar findings.

**Table 3:** Associations with RT-qPCR Ct value among 3,347 Alpha RT-qPCR-genotyped SARS-CoV-2 infections between March 23 and November 6, 2021.

Characteristics	RT-qPCR Ct value	Univariable analysis		F-test^*^	Multivariable analysis^†^	
	Mean (SD)	β coefficient [95% CI]	p-value	p-value	β coefficient [95% CI]	p-value
Age group in years				<0.001		
10-19^‡^	26.24 (5.93)	Ref.			Ref.	
<10	27.60 (5.17)	1.36 [0.43, 2.29]	0.004		1.28 [0.50, 2.06]	0.001
20-29	28.48 (5.50)	2.24 [1.46, 3.01]	<0.001		0.20 [-0.49, 0.88]	0.577
30-39	28.12 (5.72)	1.88 [1.14, 2.62]	<0.001		0.04 [-0.63, 0.71]	0.910
40-49	27.81 (5.78)	1.57 [0.78, 2.37]	<0.001		-0.16 [-0.87, 0.55]	0.661
50-59	27.94 (5.32)	1.70 [0.69, 2.71]	0.001		-0.18 [-1.07, 0.71]	0.694
60-69	27.07 (5.33)	0.83 [-0.62, 2.28]	0.263		-1.14 [-2.38, 0.10]	0.072
70-79	24.88 (6.01)	-1.36 [-4.38, 1.66]	0.377		-2.70 [-5.23, -0.17]	0.037
80+	25.93 (3.81)	-0.30 [-4.52, 3.91]	0.887		-0.13 [-3.65, 3.40]	0.943
Sex				<0.001		
Female	26.24 (5.81)	Ref.			Ref.	
Male	28.71 (5.38)	2.47 [2.07, 2.86]	<0.001		0.74 [0.35, 1.13]	<0.001
Nationality^§^				<0.001		
Qatari	26.21 (5.58)	Ref.			Ref.	
Bangladeshi	29.34 (5.07)	3.13 [2.30, 3.96]	<0.001		1.77 [0.98, 2.56]	<0.001
Egyptian	26.45 (6.07)	0.24 [-0.87, 1.34]	0.674		0.39 [-0.57, 1.35]	0.429
Filipino	26.57 (5.76)	0.36 [-0.38, 1.10]	0.339		0.84 [0.14, 1.53]	0.019
Indian	29.57 (5.23)	3.36 [2.78, 3.95]	<0.001		1.95 [1.36, 2.55]	<0.001
Nepalese	29.44 (5.15)	3.23 [2.38, 4.08]	<0.001		2.32 [1.51, 3.12]	<0.001
Pakistani	28.08 (5.24)	1.87 [1.02, 2.72]	<0.001		0.70 [-0.09, 1.50]	0.083
Sri Lankan	27.74 (6.08)	1.53 [0.39, 2.67]	0.009		0.72 [-0.31, 1.74]	0.170
Sudanese	26.50 (5.11)	0.29 [-0.92, 1.50]	0.635		0.90 [-0.16, 1.96]	0.097
Other nationalities^¶^	27.06 (5.77)	0.85 [0.22, 1.48]	0.009		0.66 [0.09, 1.23]	0.023
Reason for RT-qPCR testing				<0.001		
Survey	28.26 (5.43)	Ref.			Ref.	
Clinical suspicion	26.39 (5.87)	-1.87 [-2.43, -1.30]	<0.001		-1.54 [-2.04, -1.04]	<0.001
Contact tracing	27.05 (5.87)	-1.21 [-1.92, -0.50]	0.001		-0.54 [-1.15, 0.08]	0.088
Healthcare routine testing	28.20 (5.77)	-0.06 [-1.17, 1.05]	0.915		-0.42 [-1.38, 0.53]	0.387
Port of entry	28.61 (5.07)	0.35 [-0.30, 1.01]	0.291		-0.17 [-0.76, 0.42]	0.568
Pre-travel	30.58 (4.45)	2.32 [1.43, 3.21]	<0.001		0.01 [-0.77, 0.80]	0.976
Individual request	29.58 (5.18)	1.33 [0.64, 2.02]	<0.001		0.38 [-0.23, 0.99]	0.221
Other	28.81 (5.13)	0.56 [-1.02, 2.13]	0.490		-1.42 [-2.80, -0.05]	0.042
RT-qPCR test study-period month				<0.001		
23 March-21 April, 2021	25.15 (5.76)	Ref.			Ref.	
22 April-21 May, 2021	27.82 (5.20)	2.67 [2.23, 3.11]	<0.001		1.95 [1.53, 2.38]	<0.001
22 May-20 June, 2021	31.55 (4.25)	6.40 [5.92, 6.87]	<0.001		4.92 [4.43, 5.41]	<0.001
21 June-20 July, 2021	30.10 (3.24)	4.95 [4.29, 5.61]	<0.001		3.75 [3.09, 4.40]	<0.001
21 July-19 August, 2021	30.94 (2.92)	5.79 [5.12, 6.46]	<0.001		4.52 [3.84, 5.21]	<0.001
20 August-18 September, 2021	32.41 (2.03)	7.26 [6.19, 8.33]	<0.001		6.06 [5.00, 7.12]	<0.001
19 September-18 October, 2021	-^**^	-^**^			-^**^	
19 October-06 November, 2021	-^**^	-^**^			-^**^	
Vaccination status				<0.001		
Unvaccinated	27.52 (5.71)	Ref.			Ref.	
One dose	28.32 (5.56)	0.80 [0.06, 1.54]	0.034		1.37 [0.73, 2.00]	<0.001
Two doses						
<3 months before the RT-qPCR test	30.47 (4.63)	2.95 [2.24, 3.67]	<0.001		1.54 [0.91, 2.18]	<0.001
3-<6 months before the RT-qPCR test	30.61 (3.40)	3.10 [1.90, 4.29]	<0.001		1.14 [0.04, 2.25]	0.042
6-<9 months before the RT-qPCR test	33.89 (2.36)	6.38 [0.06, 12.69]	0.048		5.10 [-0.32, 10.52]	0.065
≥9 months before the RT-qPCR test	-^**^	-^**^			-^**^	
Three doses						
≤1 month before the RT-qPCR test	29.24 (-)	1.73 [-9.21, 12.66]	0.757		-4.05 [-13.26, 5.15]	0.388
>1 month before the RT-qPCR test	-^**^	-^**^			-^**^	
Prior SARS-CoV-2 infection				<0.001		
Never	27.04 (5.88)	Ref.			Ref.	
<90 days before the study RT-qPCR test^††^	30.50 (3.79)	3.46 [3.02, 3.90]	<0.001		3.07 [2.66, 3.49]	<0.001
Prior infection^‡‡^	32.05 (3.14)	5.01 [3.19, 6.83]	<0.001		1.83 [0.24, 3.42]	0.024

CI, confidence interval; Ct, cycle threshold; RT-qPCR, real-time reverse-transcription polymerase chain reaction; Ref., reference; SARS-CoV-2, severe acute respiratory syndrome coronavirus 2; SD, standard deviation.

^*^The two-tailed F-test of the univariable analysis.

^†^RT-qPCR Ct value was adjusted for age-group, sex, nationality, reason for RT-qPCR test, RT-qPCR test study-period month, vaccination status, and prior SARS-CoV-2 infection.

**^‡^** The 10-19 age group was chosen as a reference, and not the <10 age group, because of the different manifestations of this infection in small children.

^§^Nationalities were chosen to represent the most populous groups on Qatar.

^¶^These comprise 63 other nationalities in Qatar.

^**^Inestimable quantity due to very small number of observations.

^††^An RT-qPCR-positive test that occurred <90 days before the study RT-qPCR-positive test was included as a separate category in the analysis and was not considered a prior infection. This RT-qPCR-positive test and the study RT-qPCR-positive test may both reflect the same prolonged infection.

**^‡‡^** Prior infection was defined as an RT-qPCR-positive test that occurred ≥90 days before the RT-qPCR-positive test that is included in the study.

**Table 4:** Associations with RT-qPCR Ct value among 5,576 Beta RT-qPCR-genotyped SARS-CoV-2 infections between March 23 and November 6, 2021.

Characteristics	RT-qPCR Ct value	Univariable analysis		F-test^*^	Multivariable analysis^†^	
	Mean (SD)	β coefficient [95% CI]	p-value	p-value	β coefficient [95% CI]	p-value
Age group in years				<0.001		
10-19^‡^	23.53 (5.25)	Ref.			Ref.	
<10	25.91 (5.02)	2.38 [1.72, 3.04]	<0.001		1.80 [1.22, 2.38]	<0.001
20-29	23.56 (5.24)	0.04 [-0.50, 0.58]	0.892		-0.00 [-0.50, 0.49]	0.991
30-39	23.40 (5.09)	-0.13 [-0.63, 0.38]	0.623		-0.10 [-0.58, 0.37]	0.666
40-49	23.63 (5.05)	0.10 [-0.44, 0.65]	0.705		0.18 [-0.33, 0.69]	0.495
50-59	23.93 (5.01)	0.40 [-0.26, 1.06]	0.235		0.18 [-0.42, 0.79]	0.549
60-69	23.52 (4.29)	-0.01 [-0.98, 0.97]	0.988		-0.21 [-1.08, 0.66]	0.629
70-79	22.59 (5.51)	-0.93 [-2.78, 0.92]	0.323		-1.21 [-2.83, 0.41]	0.143
80+	23.36 (5.43)	-0.17 [-2.98, 2.64]	0.904		-1.26 [-3.71, 1.19]	0.314
Sex				0.020		
Female	23.49 (5.15)	Ref.			Ref.	
Male	23.83 (5.13)	0.34 [0.05, 0.63]	0.020		0.32 [0.04, 0.60]	0.023
Nationality^§^				0.132		
Qatari	23.88 (5.28)	Ref.			Ref.	
Bangladeshi	23.80 (4.98)	-0.08 [-0.66, 0.50]	0.790		0.64 [0.09, 1.19]	0.023
Egyptian	23.86 (5.08)	-0.02 [-0.69, 0.65]	0.950		0.67 [0.08, 1.26]	0.026
Filipino	23.42 (5.02)	-0.46 [-1.00, 0.08]	0.098		0.68 [0.18, 1.19]	0.008
Indian	23.81 (5.20)	-0.07 [-0.51, 0.38]	0.771		0.62 [0.19, 1.05]	0.005
Nepalese	23.37 (5.15)	-0.50 [-1.09, 0.08]	0.090		0.53 [-0.03, 1.09]	0.064
Pakistani	24.34 (5.21)	0.46 [-0.24, 1.15]	0.197		0.45 [-0.18, 1.07]	0.159
Sri Lankan	23.17 (4.90)	-0.71 [-1.47, 0.06]	0.071		0.22 [-0.48, 0.92]	0.540
Sudanese	23.09 (4.61)	-0.79 [-1.82, 0.24]	0.133		0.39 [-0.51, 1.29]	0.401
Other nationalities^¶^	23.72 (5.16)	-0.16 [-0.64, 0.31]	0.506		0.43 [0.00, 0.86]	0.048
Reason for RT-qPCR testing				<0.001		
Survey	24.30 (4.85)	Ref.			Ref.	
Clinical suspicion	22.78 (4.89)	-1.52 [-1.89, -1.14]	<0.001		-1.47 [-1.81, -1.14]	<0.001
Contact tracing	23.46 (5.03)	-0.84 [-1.31, -0.36]	0.001		-0.68 [-1.11, -0.25]	0.002
Healthcare routine testing	23.45 (4.93)	-0.85 [-1.59, -0.12]	0.023		-1.44 [-2.10, -0.78]	<0.001
Port of entry	27.24 (5.46)	2.94 [2.29, 3.60]	<0.001		0.35 [-0.27, 0.97]	0.268
Pre-travel	26.82 (5.25)	2.52 [1.79, 3.24]	<0.001		0.55 [-0.12, 1.22]	0.108
Individual request	24.14 (5.15)	-0.16 [-0.67, 0.35]	0.549		-0.25 [-0.71, 0.21]	0.293
Other	24.86 (6.00)	0.56 [-0.87, 2.00]	0.442		-2.19 [-3.48, -0.91]	0.001
RT-qPCR test study-period month				<0.001		
23 March-21 April, 2021	22.79 (4.73)	Ref.			Ref.	
22 April-21 May, 2021	23.16 (4.70)	0.37 [0.07, 0.67]	0.016		0.15 [-0.13, 0.44]	0.297
22 May-20 June, 2021	26.81 (5.64)	4.02 [3.40, 4.65]	<0.001		3.13 [2.53, 3.74]	<0.001
21 June-20 July, 2021	28.73 (3.86)	5.94 [5.06, 6.81]	<0.001		4.65 [3.80, 5.50]	<0.001
21 July-19 August, 2021	30.48 (2.85)	7.69 [7.16, 8.21]	<0.001		6.41 [5.82, 7.00]	<0.001
20 August-18 September, 2021	30.13 (3.66)	7.34 [6.36, 8.33]	<0.001		6.07 [5.09, 7.05]	<0.001
19 September-18 October, 2021	-^**^	-^**^			-^**^	
19 October-06 November, 2021	-^**^	-^**^			-^**^	
Vaccination status				<0.001		
Unvaccinated	23.59 (5.08)	Ref.			Ref.	
One dose	23.21 (4.91)	-0.38 [-0.82, 0.06]	0.091		0.34 [-0.05, 0.73]	0.087
Two doses						
<3 months before the RT-qPCR test	24.26 (5.31)	0.67 [0.18, 1.16]	0.008		0.68 [0.23, 1.13]	0.003
3-<6 months before the RT-qPCR test	29.16 (4.58)	5.57 [4.60, 6.54]	<0.001		0.48 [-0.47, 1.43]	0.325
6-<9 months before the RT-qPCR test	30.28 (4.50)	6.69 [2.93, 10.45]	<0.001		0.06 [-3.28, 3.40]	0.973
≥9 months before the RT-qPCR test	-^**^	-^**^			-^**^	
Three doses						
≤1 month before the RT-qPCR test	25.13 (2.72)	1.54 [-5.49, 8.57]	0.668		2.39 [-3.76, 8.53]	0.446
>1 month before the RT-qPCR test	-^**^	-^**^			-^**^	
Prior SARS-CoV-2 infection				<0.001		
Never	23.19 (5.01)	Ref.			Ref.	
<90 days before the study RT-qPCR test^††^	27.82 (4.17)	4.63 [4.21, 5.04]	<0.001		4.14 [3.75, 4.53]	<0.001
Prior infection^‡‡^	25.83 (5.31)	2.64 [1.38, 3.89]	<0.001		2.04 [0.91, 3.17]	<0.001

CI, confidence interval; Ct, cycle threshold; RT-qPCR, real-time reverse-transcription polymerase chain reaction; Ref., reference; SARS-CoV-2, severe acute respiratory syndrome coronavirus 2; SD, standard deviation.

^*^The two-tailed F-test of the univariable analysis.

^†^RT-qPCR Ct value was adjusted for age-group, sex, nationality, reason for RT-qPCR test, RT-qPCR test study-period month, vaccination status, and prior SARS-CoV-2 infection.

**^‡^** The 10-19 age group was chosen as a reference, and not the <10 age group, because of the different manifestations of this infection in small children.

^§^Nationalities were chosen to represent the most populous groups on Qatar.

^¶^These comprise 60 other nationalities in Qatar.

^**^Inestimable quantity due to very small number of observations.

^††^An RT-qPCR-positive test that occurred <90 days before the study RT-qPCR-positive test was included separately in the analysis, but was not considered a prior infection. This RT-qPCR-positive test and the study RT-qPCR-positive test may both reflect the same prolonged infection.

**^‡‡^** Prior infection was defined as an RT-qPCR-positive test that occurred ≥90 days before the RT-qPCR-positive test that is included in the study.

**Table 5:** Associations with RT-qPCR Ct value among 9,432 Delta RT-qPCR-genotyped SARS-CoV-2 infections between March 23 and November 6, 2021.

Characteristics	RT-qPCR Ct value	Univariable analysis		F-test^*^	Multivariable analysis^†^	
	Mean (SD)	β coefficient [95% CI]	p-value	p-value	β coefficient [95% CI]	p-value
Age group in years				<0.001		
10-19^‡^	22.45 (5.07)	Ref.			Ref.	
<10	24.89 (4.91)	2.44 [2.04, 2.84]	<0.001		2.45 [2.07, 2.83]	<0.001
20-29	21.90 (5.12)	-0.55 [-0.91, -0.18]	0.003		-0.54 [-0.91, -0.16]	0.005
30-39	21.93 (5.15)	-0.52 [-0.86, -0.18]	0.003		-0.64 [-1.00, -0.28]	<0.001
40-49	21.53 (5.24)	-0.92 [-1.30, -0.54]	<0.001		-1.00 [-1.39, -0.61]	<0.001
50-59	21.75 (4.95)	-0.70 [-1.20, -0.20]	0.006		-0.84 [-1.34, -0.34]	0.001
60-69	22.01 (5.28)	-0.44 [-1.17, 0.29]	0.239		-0.61 [-1.33, 0.10]	0.093
70-79	22.16 (4.76)	-0.29 [-1.73, 1.16]	0.696		-0.04 [-1.42, 1.33]	0.952
80+	21.39 (5.29)	-1.06 [-3.37, 1.26]	0.371		-0.76 [-2.94, 1.42]	0.493
Sex				<0.001		
Female	21.92 (5.12)	Ref.			Ref.	
Male	22.58 (5.26)	0.66 [0.44, 0.87]	<0.001		0.48 [0.27, 0.70]	<0.001
Nationality^§^				<0.001		
Qatari	22.19 (4.95)	Ref.			Ref.	
Bangladeshi	21.75 (5.63)	-0.44 [-1.01, 0.13]	0.129		-0.14 [-0.71, 0.42]	0.617
Egyptian	22.88 (5.22)	0.68 [0.24, 1.13]	0.002		-0.08 [-0.50, 0.35]	0.717
Filipino	21.30 (5.46)	-0.89 [-1.35, -0.44]	<0.001		-0.37 [-0.83, 0.08]	0.106
Indian	22.21 (5.27)	0.02 [-0.30, 0.34]	0.900		-0.29 [-0.64, 0.06]	0.110
Nepalese	22.50 (5.17)	0.31 [-0.24, 0.86]	0.265		-0.12 [-0.68,0.44]	0.677
Pakistani	22.50 (5.29)	0.30 [-0.19, 0.80]	0.227		0.07 [-0.41, 0.56]	0.766
Sri Lankan	23.21 (5.54)	1.02 [0.20, 1.83]	0.015		0.33 [-0.44, 1.10]	0.399
Sudanese	22.89 (4.85)	0.70 [-0.06, 1.46]	0.073		0.87 [0.16, 1.58]	0.017
Other nationalities^¶^	22.51 (5.25)	0.32 [0.02, 0.61]	0.034		0.13 [-0.15, 0.42]	0.357
Reason for RT-qPCR testing				<0.001		
Survey	22.44 (5.08)	Ref.			Ref.	
Clinical suspicion	21.16 (4.99)	-1.28 [-1.61, -0.94]	<0.001		-1.36 [-1.69, -1.04]	<0.001
Contact tracing	22.25 (5.15)	-0.19 [-0.60, 0.23]	0.371		-0.37 [-0.76, 0.03]	0.073
Healthcare routine testing	22.71 (5.26)	0.27 [-0.49, 1.04]	0.483		-0.17 [-0.90, 0.56]	0.650
Port of entry	22.94 (5.18)	0.50 [0.16, 0.85]	0.004		0.93 [0.59, 1.27]	<0.001
Pre-travel	24.14 (5.27)	1.70 [1.25, 2.15]	<0.001		1.80 [1.37, 2.22]	<0.001
Individual request	22.15 (5.41)	-0.29 [-0.79, 0.21]	0.262		0.36 [-0.14, 0.86]	0.158
Other	25.20 (5.18)	2.76 [1.63, 3.89]	<0.001		-0.01 [-1.11, 1.09]	0.989
RT-qPCR test study-period month				<0.001		
23 March-21 April, 2021	21.55 (4.55)	Ref.			Ref.	
22 April-21 May, 2021	21.54 (4.89)	-0.02 [-0.85, 0.81]	0.968		-0.17 [-0.96, 0.61]	0.666
22 May-20 June, 2021	22.64 (5.69)	1.09 [0.25, 1.93]	0.011		1.05 [0.24, 1.86]	0.011
21 June-20 July, 2021	20.34 (4.44)	-1.22 [-1.99, -0.44]	0.002		-1.14 [-1.90, -0.38]	0.003
21 July-19 August, 2021	21.90 (5.07)	0.35 [-0.38, 1.07]	0.348		0.13 [-0.58, 0.84]	0.722
20 August-18 September, 2021	23.47 (5.41)	1.92 [1.19, 2.65]	<0.001		1.55 [0.84, 2.27]	<0.001
19 September-18 October, 2021	23.04 (5.20)	1.49 [0.72, 2.26]	<0.001		1.23 [0.47, 1.98]	0.001
19 October-06 November, 2021	21.76 (4.85)	0.21 [-0.59, 1.01]	0.606		0.26 [-0.53, 1.05]	0.524
Vaccination status				<0.001		
Unvaccinated	22.56 (5.31)	Ref.			Ref.	
One dose	21.22 (5.24)	-1.34 [-2.02, -0.66]	<0.001		0.35 [-0.29, 0.99]	0.281
Two doses						
<3 months before the RT-qPCR test	22.26 (4.98)	-0.30 [-0.72, 0.11]	0.151		0.87 [0.47, 1.27]	<0.001
3-<6 months before the RT-qPCR test	21.98 (5.04)	-0.59 [-0.84, -0.33]	<0.001		0.22 [-0.06, 0.50]	0.120
6-<9 months before the RT-qPCR test	21.62 (4.97)	-0.94 [-1.40, -0.49]	<0.001		-0.23 [-0.72, 0.25]	0.347
≥9 months before the RT-qPCR test	18.81 (2.22)	-3.75 [-8.85, 1.35]	0.149		-3.15 [-7.87, 1.57]	0.191
Three doses						
≤1 month before the RT-qPCR test	17.72 (2.35)	-4.84 [-12.05, 2.36]	0.188		-3.46 [-10.16, 3.23]	0.311
>1 month before the RT-qPCR test	19.80 (2.97)	-2.77 [-7.87, 2.33]	0.287		-1.18 [-5.89, 3.53]	0.623
Prior SARS-CoV-2 infection				<0.001		
Never	21.93 (5.11)	Ref.			Ref.	
<90 days before the study RT-qPCR test^††^	25.32 (4.95)	3.39 [3.05, 3.72]	<0.001		4.03 [3.69, 4.38]	<0.001
Prior infection^‡‡^	24.00 (5.64)	2.07 [1.13, 3.02]	<0.001		2.15 [1.26, 3.04]	<0.001

CI, confidence interval; Ct, cycle threshold; RT-qPCR, real-time reverse-transcription polymerase chain reaction; Ref., reference; SARS-CoV-2, severe acute respiratory syndrome coronavirus 2; SD, standard deviation.

^*^The two-tailed F-test of the univariable analysis.

^†^RT-qPCR Ct value was adjusted for age-group, sex, nationality, reason for RT-qPCR test, RT-qPCR test study-period month, vaccination status, and prior SARS-CoV-2 infection.

**^‡^** The 10-19 age group was chosen as a reference, and not the <10 age group, because of the different manifestations of this infection in small children.

^§^Nationalities were chosen to represent the most populous groups on Qatar.

^¶^These comprise 87 other nationalities in Qatar.

^††^An RT-qPCR-positive test that occurred <90 days before the study RT-qPCR-positive test was included as a separate category in the analysis and was not considered a prior infection. This RT-qPCR-positive test and the study RT-qPCR-positive test may both reflect the same prolonged infection.

**^‡‡^** Prior infection was defined as an RT-qPCR-positive test that occurred ≥90 days before the RT-qPCR-positive test that is included in the study.

## Discussion

Delta infections were associated with considerably lower Ct values than Beta infections, indicating higher infectiousness of this variant, perhaps because of higher viral load and/or longer duration of infection. This appears to be the first direct comparison of the infectiousness of Delta versus Beta infections, and supports the high infectiousness of the Delta variant compared to pre-Omicron variants such as Alpha, as reported previously (33, 34). This difference in viral load between Delta and Beta appears also to extend to severity of infection, as Delta infections were found associated with higher severity than Beta infections (35). Of note, Beta infections were also found earlier to be associated with higher severity than Alpha infections (36). Worth mentioning is that factors other than viral load, such as the variations in the spike proteins or any other differences in the biological properties of the viruses, may also affect the infectiousness of different SARS-CoV-2 variants (37).

Prior immunity against SARS-CoV-2 infection, whether due to vaccination or prior infection, was associated with higher Ct value at infection, and thus lower infectiousness of breakthrough infections. This confirms earlier findings (15, 16), and suggests that strength of immunity is manifest not only in protection against infection, but also against the infectiousness, if a breakthrough infection occurs (15). However, this effect appeared to depend on the time since the prior immunological event (**Table 2**). Ct values decreased gradually with time since second-dose vaccination, paralleling the established pattern of waning of vaccine effectiveness after the second dose (6, 38, 39).

Ct values decreased with age, perhaps reflecting slower virus clearance with aging (40) and confirming our earlier findings (16). Ct values were particularly high for infections among small children <10 years of age. This finding supports a lesser role for small children than adults in the transmission of infection, as suggested in studies of secondary transmission within households (41–43). There were differences in Ct value by sex and nationality, but these may be a consequence of different test-seeking behaviors for different socio-economic groups in Qatar’s diverse population. Ct values also varied by reason for testing, with lower Ct values of infections diagnosed because of suspicion of infection, and higher Ct values of infections diagnosed through routine testing unrelated to infection exposure. This finding also confirms our earlier finding for Omicron infections (16).

The study has limitations. RT-qPCR genotyping in Qatar started only after the Alpha wave peaked in the first week of March 2021 and thus many Alpha infections may have been older infections explaining the relatively higher Ct values of these infections compared to Delta or Beta infections. The study included only documented RT-qPCR-confirmed infections. It is possible that infections in those with a prior infection or those vaccinated are less likely to be diagnosed, perhaps because of minimal or no symptoms (15). Nevertheless, RT-qPCR testing in Qatar is done at a mass scale, where a significant proportion of the population is being tested weekly (6). The majority of infections are identified *via* routine testing and not because of appearance of symptoms (**Table 1**) (6).

Other limitations include the small number of individuals who received the booster dose by the end of the study precluding the estimation of effect of booster vaccination on Ct value and the inability to factor in the duration between symptom onset and RT-qPCR test (for symptomatic cases) as the date of symptom onset was not available. The study population consisted mostly of working-age adults; thus, the results may not be generalizable to the elderly.

In conclusion, the Delta variant appears substantially more infectious than the Beta variant, explaining its global reach in the pre-Omicron era. Infectiousness of SARS-CoV-2 infections increases with age, apparently reflecting slower virus clearance with aging. Prior immunity against SARS-CoV-2 infection, whether due to vaccination or prior infection, is associated with lower infectiousness of breakthrough infections. However, infectiousness of breakthrough infections increases gradually with time since second-dose vaccination, paralleling the waning of vaccine effectiveness after the second dose. These scientific insights enhance our understanding of the evolving epidemiology of the virus with implications for appropriate public health response against its different variants.

## Data availability statement

The datasets presented in this article are not readily available because these are properties of the Qatar Ministry of Public Health that were provided to the researchers through a restricted-access agreement that prevents sharing the datasets with a third party or publicly. The data are available under restricted access for confidentiality. The raw data are protected and are not available due to data privacy laws. Aggregate data are available within the manuscript. Requests to access the datasets should be directed to Her Excellency the Minister of Public Health, https://www.moph.gov.qa/english/OurServices/eservices/Pages/Governmental-Health-Communication-Center.aspx.

## Ethics statement

The studies involving human participants were reviewed and approved by Hamad Medical Corporation and Weill Cornell Medicine-Qatar Institutional Review Boards. Written informed consent from the participants’ legal guardian/next of kin was not required to participate in this study in accordance with the national legislation and the institutional requirements.

## Author contributions

SQ co-designed the study, performed the statistical analyses, and co-wrote the first draft of the article. PT and MH conducted the multiplex, RT-qPCR variant screening and viral genome sequencing. HC co-designed the study and supported the statistical analyses. LA-R conceived and co-designed the study, led the statistical analyses, and co-wrote the first draft of the article. All authors contributed to data collection and acquisition, database development, discussion and interpretation of the results, and to the writing of the manuscript. All authors have read and approved the final manuscript.

## Acknowledgments

We acknowledge the many dedicated individuals at Hamad Medical Corporation, the Ministry of Public Health, the Primary Health Care Corporation, Qatar Biobank, Sidra Medicine, and Weill Cornell Medicine-Qatar for their diligent efforts and contributions to make this study possible. The authors are grateful for institutional salary support from the Biomedical Research Program and the Biostatistics, Epidemiology, and Biomathematics Research Core, both at Weill Cornell Medicine-Qatar, as well as for institutional salary support provided by the Ministry of Public Health, Hamad Medical Corporation, and Sidra Medicine. The authors are also grateful for the Qatar Genome Programme and Qatar University Biomedical Research Center for institutional support for the reagents needed for the viral genome sequencing. The funders of the study had no role in study design, data collection, data analysis, data interpretation, or writing of the article. Statements made herein are solely the responsibility of the authors.

## Conflict of interest

AB has received institutional grant funding from Gilead Sciences unrelated to the work presented in this paper.

The remaining authors declare that the research was conducted in the absence of any commercial or financial relationships that could be construed as a potential conflict of interest.

## Publisher’s note

All claims expressed in this article are solely those of the authors and do not necessarily represent those of their affiliated organizations, or those of the publisher, the editors and the reviewers. Any product that may be evaluated in this article, or claim that may be made by its manufacturer, is not guaranteed or endorsed by the publisher.
