# Supplementary material for: Effects of SARS-CoV-2 Alpha, Beta, and Delta variants, age, vaccination, and prior infection on infectiousness of SARS-CoV-2 infections
Source: Front Immunol. 2022 Sep 13;13:984784. doi: 10.3389/fimmu.2022.984784 (PMC9513583; doi:10.3389/fimmu.2022.984784)
Supplement: Supplementary file 1 [file DataSheet_1.docx]

**Supplementary Material**

**Table of contents**

[**1. Laboratory methods and variant ascertainment** 2](#_Toc107323813)

[**1.1 Real-time reverse-transcription polymerase chain reaction testing** 2](#_Toc107323814)

[**1.2 Classification of infections by variant type** 2](#_Toc107323815)

[**2. Supplementary tables** 4](#_Toc107323816)

[**Supplementary table 1. STROBE checklist for cross-sectional studies.** 4](#_Toc107323817)

[**References** 6](#_Toc107323818)

**1. Laboratory methods** **and variant ascertainment**

## **1.1 Real-time reverse-transcription polymerase chain reaction testing**

Nasopharyngeal and/or oropharyngeal swabs were collected for polymerase chain reaction (PCR) testing and placed in Universal Transport Medium (UTM). Aliquots of UTM were: 1) extracted on KingFisher Flex (Thermo Fisher Scientific, USA), MGISP-960 (MGI, China), or ExiPrep 96 Lite (Bioneer, South Korea) followed by testing with real-time reverse-transcription PCR (RT-qPCR) using TaqPath COVID-19 Combo Kits (Thermo Fisher Scientific, USA) on an ABI 7500 FAST (Thermo Fisher Scientific, USA); 2) tested directly on the Cepheid GeneXpert system using the Xpert Xpress SARS-CoV-2 (Cepheid, USA); or 3) loaded directly into a Roche cobas 6800 system and assayed with the cobas SARS-CoV-2 Test (Roche, Switzerland). The first assay targets the viral S, N, and ORF1ab gene regions. The second targets the viral N and E-gene regions, and the third targets the ORF1ab and E-gene regions.

All PCR testing was conducted at the Hamad Medical Corporation Central Laboratory or Sidra Medicine Laboratory, following standardized protocols. All PCR testing was performed with extensively used, investigated, and validated commercial platforms having essentially 100% sensitivity and specificity.

## **1.2 Classification of infections by variant type**

Surveillance for SARS-CoV-2 variants in Qatar is mainly based on viral genome sequencing and multiplex RT-qPCR variant screening(1) of random positive clinical samples,(2-7) complemented by deep sequencing of wastewater samples.(4, 8)

The accuracy of the RT-qPCR genotyping was verified against either Sanger sequencing of the receptor-binding domain (RBD) of SARS-CoV-2 surface glycoprotein (S) gene, or by viral whole-genome sequencing on a Nanopore GridION sequencing device. From 236 random samples (27 Alpha-like, 186 Beta-like, and 23 “other” variants), PCR genotyping results for Alpha-like, Beta-like, and ‘other’ variants were in 88.8% (23 out of 27), 99.5% (185 out of 186), and 100% (23 out of 23) agreement with the SARS-CoV-2 lineages assigned by sequencing.

Within the “other” variant category, Sanger sequencing and/or Illumina sequencing of the RBD of SARS-CoV-2 spike gene on 728 random samples confirmed that 701 (96.3%) were Delta cases and 17 (2.3%) were other variant cases, with 10 (1.4%) samples failing lineage assignment.^6,8^ Accordingly, a Delta case was proxied as any “other” case identified through the RT-qPCR based variant screening.

All the variant RT-qPCR screening was conducted at the Sidra Medicine Laboratory following standardized protocols.

**2. Supplementary tables**

**Supplementary table 1. STROBE checklist for cross-sectional studies.**

|  | **Item No** | **Recommendations** | **Main text page No** |
| --- | --- | --- | --- |
| **Title and abstract** | 1 | (*a*) Indicate the study’s design with a commonly used term in the title or the abstract | Methods (‘Study population, data sources, and study design’, paragraph 1) |
|  |  | (*b*) Provide in the abstract an informative and balanced summary of what was done and what was found | Abstract |
| **Introduction** | | |  |
| Background/rationale | 2 | Explain the scientific background and rationale for the investigation being reported | Introduction |
| Objectives | 3 | State specific objectives, including any prespecified hypotheses | Methods (‘Study population, data sources, and study design’) |
| **Methods** | | |  |
| Study design | 4 | Present key elements of study design early in the paper | Methods (‘Study population, data sources, and study design’) |
| Setting | 5 | Describe the setting, locations, and relevant dates, including periods of recruitment, exposure, follow-up, and data collection | Methods (‘Study population, data sources, and study design’) and Section 1 (‘Classification of infections by variant type’) of Supplementary Material |
| Participants | 6 | (*a*) Give the eligibility criteria, and the sources and methods of selection of participants | Methods (‘Study population, data sources, and study design’) & Figure 1 |
| Variables | 7 | Clearly define all outcomes, exposures, predictors, potential confounders, and effect modifiers. Give diagnostic criteria, if applicable | Methods (‘Study population, data sources, and study design’ & ‘Statistical analysis’) & Sections 1 (‘Classification of infections by variant type’) of Supplementary Material |
| Data sources/ measurement | 8 | For each variable of interest, give sources of data and details of methods of assessment (measurement). Describe comparability of assessment methods if there is more than one group | Methods (‘Study population, data sources, and study design’), Table 1, & Section 1 of Supplementary Material |
| Bias | 9 | Describe any efforts to address potential sources of bias | Methods (‘Study population, data sources, and study design’ & ‘Statistical analysis’) |
| Study size | 10 | Explain how the study size was arrived at | Methods (‘Study population, data sources, and study design’) & Figure 1 |
| Quantitative variables | 11 | Explain how quantitative variables were handled in the analyses. If applicable, describe which groupings were chosen and why | Methods (‘Study population, data sources, and study design’ & ‘Statistical analysis’), & Tables 1-5 |
| Statistical methods | 12 | (*a*) Describe all statistical methods, including those used to control for confounding | Methods (‘Statistical analysis’) |
|  |  | (*b*) Describe any methods used to examine subgroups and interactions | Methods (‘Study population, data sources, and study design’ & ‘Statistical analysis’), & Tables 3-5 |
|  |  | (*c*) Explain how missing data were addressed | Methods (‘Study population, data sources, and study design’) & Figure 1 |
|  |  | (*d*) If applicable, describe analytical methods taking account of sampling strategy | NA |
|  |  | (*e*) Describe any sensitivity analyses | NA |
| **Results** | | |  |
| Participants | 13 | (a) Report numbers of individuals at each stage of study—eg numbers potentially eligible, examined for eligibility, confirmed eligible, included in the study, completing follow-up, and analysed | Figure 1 |
|  |  | (b) Give reasons for non-participation at each stage |  |
|  |  | (c) Consider use of a flow diagram |  |
| Descriptive data | 14 | (a) Give characteristics of study participants (eg demographic, clinical, social) and information on exposures and potential confounders | Table 1 |
|  |  | (b) Indicate number of participants with missing data for each variable of interest | Methods (‘Study population, data sources, and study design’) & Figure 1 |
| Outcome data | 15 | Report numbers of outcome events or summary measures | Results & Table 2 |
| Main results | 16 | (*a*) Give unadjusted estimates and, if applicable, confounder-adjusted estimates and their precision (eg, 95% confidence interval). Make clear which confounders were adjusted for and why they were included | Results & Table 2 |
|  |  | (*b*) Report category boundaries when continuous variables were categorized | Tables 1-2 |
|  |  | (*c*) If relevant, consider translating estimates of relative risk into absolute risk for a meaningful time period | NA |
| Other analyses | 17 | Report other analyses done—eg analyses of subgroups and interactions, and sensitivity analyses | Results & Tables 3-5 |
| **Discussion** | | |  |
| Key results | 18 | Summarise key results with reference to study objectives | Discussion, paragraph 1 |
| Limitations | 19 | Discuss limitations of the study, taking into account sources of potential bias or imprecision. Discuss both direction and magnitude of any potential bias | Discussion, paragraph 4-5 |
| Interpretation | 20 | Give a cautious overall interpretation of results considering objectives, limitations, multiplicity of analyses, results from similar studies, and other relevant evidence | Discussion, paragraph 1 |
| Generalisability | 21 | Discuss the generalisability (external validity) of the study results | Discussion, paragraph 5 |
| **Other information** | | |  |
| Funding | 22 | Give the source of funding and the role of the funders for the present study and, if applicable, for the original study on which the present article is based | Acknowledgements |

Abbreviations: NA: not applicable.

# **References**

1. Vogels C, Fauver J, Grubaugh N. Multiplexed Rt-Qpcr to Screen for Sars-Cov-2 B.1.1.7, B.1.351, and P.1 Variants of Concern V.3. Dx.Doi.Org/10.17504/Protocols.Io.Br9vm966 (2021) [June 6, 2021]. Available from: <https://www.protocols.io/view/multiplexed-rt-qpcr-to-screen-for-sars-cov-2-b-1-1-br9vm966>.

2. Abu-Raddad LJ, Chemaitelly H, Butt AA, National Study Group for Covid Vaccination. Effectiveness of the Bnt162b2 Covid-19 Vaccine against the B.1.1.7 and B.1.351 Variants. *N Engl J Med* (2021) 385(2):187-9. Epub 2021/05/06. doi: 10.1056/NEJMc2104974.

3. Chemaitelly H, Yassine HM, Benslimane FM, Al Khatib HA, Tang P, Hasan MR, et al. Mrna-1273 Covid-19 Vaccine Effectiveness against the B.1.1.7 and B.1.351 Variants and Severe Covid-19 Disease in Qatar. *Nat Med* (2021) 27(9):1614-21. Epub 2021/07/11. doi: 10.1038/s41591-021-01446-y.

4. National Project of Surveillance for Variants of Concern and Viral Genome Sequencing. Qatar Viral Genome Sequencing Data. Data on Randomly Collected Samples. <Https://Www.Gisaid.Org/Phylodynamics/Global/Nextstrain/> (2021). Available from: <https://www.gisaid.org/phylodynamics/global/nextstrain/>.

5. Benslimane FM, Al Khatib HA, Al-Jamal O, Albatesh D, Boughattas S, Ahmed AA, et al. One Year of Sars-Cov-2: Genomic Characterization of Covid-19 Outbreak in Qatar. *Front Cell Infect Microbiol* (2021) 11:768883. Epub 2021/12/07. doi: 10.3389/fcimb.2021.768883.

6. Hasan MR, Kalikiri MKR, Mirza F, Sundararaju S, Sharma A, Xaba T, et al. Real-Time Sars-Cov-2 Genotyping by High-Throughput Multiplex Pcr Reveals the Epidemiology of the Variants of Concern in Qatar. *Int J Infect Dis* (2021) 112:52-4. Epub 2021/09/16. doi: 10.1016/j.ijid.2021.09.006.

7. Chemaitelly H, Tang P, Hasan MR, AlMukdad S, Yassine HM, Benslimane FM, et al. Waning of Bnt162b2 Vaccine Protection against Sars-Cov-2 Infection in Qatar. *N Engl J Med* (2021) 385(24):e83. Epub 2021/10/07. doi: 10.1056/NEJMoa2114114.

8. Saththasivam J, El-Malah SS, Gomez TA, Jabbar KA, Remanan R, Krishnankutty AK, et al. Covid-19 (Sars-Cov-2) Outbreak Monitoring Using Wastewater-Based Epidemiology in Qatar. *Sci Total Environ* (2021) 774:145608. Epub 2021/02/20. doi: 10.1016/j.scitotenv.2021.145608.
